# Integration of circadian rhythms and immunotherapy for enhanced precision in brain cancer treatment

**DOI:** 10.1016/j.ebiom.2024.105395

**Published:** 2024-10-15

**Authors:** Matthias Quist, Maas van Os, Linda W. van Laake, Niels Bovenschen, Sandra Crnko

**Affiliations:** a Department of Pathology, University Medical Centre Utrecht, Utrecht University, Utrecht, the Netherlands; b Department of Cardiology, Experimental Cardiology Laboratory, University Medical Centre Utrecht, Utrecht, the Netherlands; c Regenerative Medicine Centre and Circulatory Health Research Centre, University Medical Centre Utrecht, Utrecht, the Netherlands; d Centre for Translational Immunology, University Medical Centre Utrecht, Utrecht University, Utrecht, the Netherlands

**Keywords:** Circadian rhythms, Brain cancer, Immune system, Immunotherapy, Immune checkpoints, Adoptive cellular therapy, Chronomodulated therapy

## Abstract

Circadian rhythms significantly impact (patho)physiological processes, with disruptions linked to neurodegenerative diseases and heightened cancer vulnerability. While immunotherapy has shown promise in treating various cancers, its efficacy in brain malignancies remains limited. This review explores the nexus of circadian rhythms and immunotherapy in brain cancer treatment, emphasising precision through alignment with the body's internal clock. We evaluate circadian regulation of immune responses, including cell localisation and functional phenotype, and discuss how circadian dysregulation affects anti-cancer immunity. Additionally, we analyse and assess the effectiveness of current immunotherapeutic approaches for brain cancer including immune checkpoint blockades, adoptive cellular therapies, and other novel strategies. Future directions, such as chronotherapy and personalised treatment schedules, are proposed to optimise immunotherapy precision against brain cancers. Overall, this review provides an understanding of the often-overlooked role of circadian rhythms in brain cancer and suggests avenues for improving immunotherapeutic outcomes.

**Search strategy and selection criteria**

A literature search was performed on PubMed with the following search terms (ependymoma [tiab] OR atypical teratoma [tiab] OR glioma [tiab] OR glioblastoma [tiab] OR pineal tum∗ [tiab] OR pituitary adenoma [tiab] OR meningioma [tiab] OR medulloblastoma [tiab] OR astrocytoma [tiab] OR oligodendrocytoma [tiab] OR craniopharyngioma [tiab] OR brain cancer [tiab]) AND (circadian [tiab] OR clock prot∗ [tiab] OR circadian rhythms [tiab] OR circadian clocks [tiab] OR molecular clock [tiab] OR BMAL1 [tiab] OR CLOCK [tiab] OR CRY1 [tiab] OR CRY2 [tiab] OR CRY3 [tiab] OR PER1 [tiab] OR PER2 [tiab] OR RORα [tiab] OR RORβ[tiab] OR RORγ [tiab] OR REV-ERBα [tiab] OR REV-ERBβ [tiab]) from January 2015 until June 2024. Only original research papers published in English were reviewed. The final reference list was selected based on originality and relevance to the scope of this review.

## Introduction

In 2017 Jeffrey C. Hall, Michael Rosbash and Michael W. Young were awarded with the Nobel Prize for their discovery of the molecular components that comprise the biological clock.^1^, ^2^, ^3^, ^4^ Their research was pivotal in the uncovering of the role of circadian rhythms in physiology and pathophysiology. Nowadays, it is established that circadian (Latin “circa diem” = about a day) rhythms are an outward manifestation of an internal timing system, which is crucial for maintaining homeostatic balance in organisms.

**Fig. 1:**
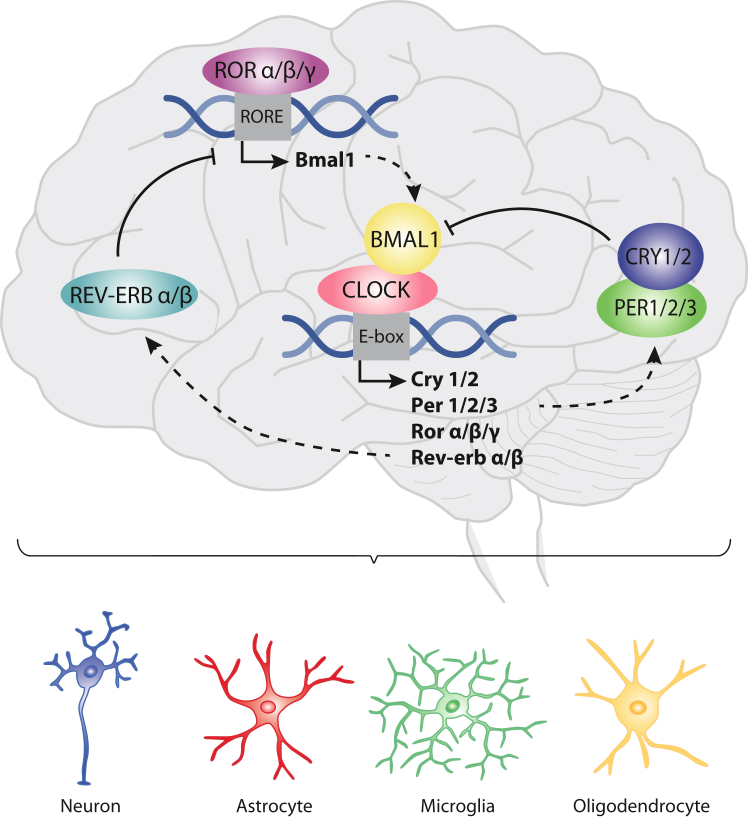
**Overview of the core clock machinery in the brain.** The transcriptional activators circadian locomotor output cycles kaput (CLOCK) and brain and muscle ARNT-Like 1 (BMAL1) form heterodimers that bind to enhancer box (E-box) elements, thereby driving the transcription of *cryptochrome (CRY1/2), period (PER1/2/3)* and tyrosine-protein kinase transmembrane receptor *RORα/β/γ* and *REV-ERBα/β*.^5^^,^^6^ PER/CRY heterodimers accumulate to inhibit BMAL1/CLOCK activity. Concurrently, RORα/β/γ and REV-ERBα/β compete for binding to ROR response element (RORE) on the *BMAL1* promoter, respectively activating or inhibiting its transcription. This intricate feedback loop generates rhythmic fluctuations in clock-controlled gene expression within 24-h cycles.^5^^,^^7^ The molecular clock is present in virtually all cell types in the human body, including neurons, astrocytes, microglia, and oligodendrocytes and drives daily fluctuations in their morphology and functionality.^8^

Circadian rhythms at the molecular level are generated by a core molecular clock, which consists of an evolutionarily conserved transcriptional-translational feedback loop (TTFL; Fig. 1).^5^ The molecular clock mechanism comprises intertwined positive and negative feedback loops. At the core, the transcriptional activators circadian locomotor output cycles kaput (CLOCK; and its paralog, NPAS2) and brain and muscle ARNT-Like 1 (BMAL1), form heterodimers that bind to enhancer box elements (E-boxes) of *cryptochrome (CRY1/2), period (PER1/2/3)* and tyrosine-protein kinase transmembrane receptor *RORα/β/γ* and *REV-ERBα/β*, thereby stimulating their transcription.^5^^,^^6^ Subsequently, PER/CRY heterodimers accumulate and inhibit BMAL1/CLOCK activity. RORα/β/γ and REV-ERBα/β compete for binding to ROR response element (RORE), a promotor for *BMAL1*, leading either to its activation or inhibition, respectively. Together, the interplay of these feedback loops causes rhythmic fluctuations in clock-controlled gene expression, with a period of approximately 24 h. Virtually all cell types, including neurons and glial cells, express autonomous circadian rhythms driven by this TTFL.^9^ The oscillations in expression of the circadian core clock genes, and the genes that they regulate by binding to transcription enhancing elements, drive cell functionality across the 24-h cycle.^10^ For instance, the gut clock regulates intestinal motility and nutrient absorption to match the timing of maximal monosaccharide uptake to the habitual feeding periods.^11^ Furthermore, circadian rhythms in the kidneys cause fluctuations in renal plasma flow, glomerular filtration rate and tubular reabsorption and secretion processes.^12^ To prevent misalignment of these cellular processes across the whole body, daily synchronisation of peripheral clocks is needed.

Daily synchronisation is regulated by a central master clock, located in the suprachiasmatic nucleus (SCN) of the hypothalamus.^6^ The SCN generates coherent oscillations of the molecular clock, thereby synchronising peripheral clocks.^6^ Environmental cues such as exercise, food and social contacts act as Zeitgebers (“time givers”) to align the clock of the SCN to the outside world, with light being the main clock input signal.^5^ The SCN in turn harmonises all peripheral clocks, thereby aligning cellular processes across the whole body.

Of note, officially 24-h rhythms in the presence of natural synchronisers are referred to as diurnal (or nocturnal) rhythms, while the term ‘circadian rhythms’ is used for rhythms that are expressed in the absence of any 24-h signal cues from the external environment.^13^ However, almost all diurnal rhythms are found to be circadian. Therefore, in this review the term circadian will be used for all 24-h oscillatory processes, regardless of external inputs.

Disruptions of the circadian clock can result in a range of health issues spanning neurological, metabolic, endocrine, cardiovascular, and immune system morbidities.^14^ Animal studies show that disturbances of the molecular clock in the brain can lead to spontaneous astrogliosis, increased oxidative damage, synaptic degeneration, and other neurodegenerative alterations.^15^ Moreover, these disruptions are linked to neurodegenerative disorders such as Alzheimer's Disease and Parkinson's Disease, as well as an increased risk for developing various malignancies, including brain cancers.^16^^,^^17^

Brain cancer treatment faces several challenges. Depending on the severity and invasiveness of brain cancers, treatment outcome varies greatly between different forms of brain tumours.^18^ Depending on localisation in the brain, tumours are typically challenging to remove completely during surgery, while systemic chemotherapies and precision medicine delivery is hindered by the blood–brain barrier (BBB).^19^ Furthermore, brain cancers often exhibit cellular and molecular heterogeneity, making them resistant to conventional treatments.^19^ These challenges necessitate aggressive treatment regimes, such as high-dose radiotherapy or combination chemotherapy, often culminating in severe side effects and reduced quality of life in survivors.^19^ An emerging alternative treatment is immunotherapy. These therapies stimulate the immune system to create a targeted reaction to the tumour.^20^ The immune system comprises of innate and adaptive immunity.^21^ Innate immunity offers an immediate response utilising natural killer (NK) cells and macrophages to detect and destroy abnormal cells based on stress signals and altered cell surface molecules. In the case of cancer, innate immune cells train adaptive immune cells, including T lymphocytes, to specifically target tumour cells.^21^ Although immunotherapy has shown promise in cancers like melanoma and colorectal cancer,^22^ its success in brain cancer has been limited, underscoring the need for innovative therapeutic approaches. Circadian rhythms and the molecular clock herein offer an unexplored avenue.

Components of the circadian clock in the tumour microenvironment (TME) are important regulators of cancer cell stemness, metastasis and therapy resistance.^23^ This review explores the impact of the circadian clock on brain tumour formation, the brain TME, and immunosuppression. This approach includes optimising therapy timing through chronotherapy and exploring the molecular function of the circadian clock in brain cancer. Furthermore, we will examine how the circadian clock impacts the immune system and tumour immunology, highlighting its potential to enhance the efficacy of immunotherapy. We postulate that enhancing the understanding of the complex brain tumour biology and TME, with particular emphasis on the role of circadian clock, is essential for developing effective (immuno)therapies and boosting existing ones.

## How does the molecular clock tick in the healthy central nervous system?

Oscillations of the circadian clock can be found in virtually all tissues, including glial cells, and have important functions in brain development and functionality.^8^ Given that most brain malignancies arise from glial cells or their precursors, it is of relevance to understand the role of the clock in their physiology.^24^ Therefore, in this section, the influence of the molecular clock on the functionality of various glial cell types will be described.

Astrocytes are the most abundant population of glial cells in the mammalian brain.^25^ These cells provide important metabolic and trophic support to neurons, which is crucial for nervous system health.^26^ For instance, perisynaptic astrocyte processes remove neurotransmitters such as glutamate from the interstitial space to prevent extra-synaptic accumulation and spillover to nearby synapses.^27^ Furthermore, astrocytes control the ionic balance at the synapse, sustaining proper synaptic transmission.^25^

Studies using *in vitro* cultures of mouse cortical astrocytes have shown that astrocytes exhibit rhythmic changes governed by clock genes.^28^ For example, oscillations of *Clock*, *Per1*, *Per2*, and IP_3_-dependent calcium signalling regulate the daily rhythms of ATP release in astrocytes.^29^ BMAL1 acts as a cell-autonomous regulator of astrocyte activation and neurotrophic function, while reduced levels of BMAL1 induce astrogliosis.^30^ *In vitro* experiments with primary mouse astrocytes have shown that Ror-α is upregulated during hypoxia, leading to downregulation of hypoxic inducible factor 1α.^31^ Furthermore, Clock, Per2, and Npas2 influence glutamate uptake levels, though without detectable circadian variation.^32^ Interestingly, astrocytes in the SCN of mice can modulate daily rhythms in the SCN and behaviour, and deletion of *Bmal1* in astrocytes lengthened the circadian period of rest-activity rhythms.^33^ Thus, the core circadian clock has important roles in functioning of astrocytes.

Another cell type residing in the central nervous system (CNS) are the microglia. These macrophage-like immune cells remove dead cells in both the developing brain and the adult CNS, thus aiding normal brain development.^34^ Microglia also participate in synaptic development and maintenance. Like most cells, microglia possess a circadian clock that regulates their immune activity.^35^ In rats, for instance, a circadian rhythm of TNF-α, interleukin (IL)-1β and IL-6 expression peaks during the middle of the light phase (rest phase). Disruption of clock genes in microglia can induce chronic neuroinflammation and has been associated with the early onset of Alzheimer's Disease.^36^ The clock gene expression in microglia can be affected by obesogenic diets, leading to chronic activation of microglia in rats.^37^ The molecular clock is important in balancing the microglial phenotype and preventing neuroinflammation, with Rev-erbα playing a crucial role. Low levels of Rev-erbα increase synaptic phagocytosis, while the loss of Rev-erbα function results in spontaneous neuroinflammation and neuronal dysfunction.^38^^,^^39^

Lastly, oligodendrocytes, the third group of glial cells, are crucial for signal conduction in the CNS. These cells form a myelin sheath that facilitates rapid transmission of action potentials along the axon and provide metabolic support.^40^ Unlike Schwann cells, which myelinate single axons in the peripheral nervous system, oligodendrocytes can encapsulate multiple axons. They generate from oligodendrocyte progenitor cells (OPCs) through a strictly coordinated process of maturation, proliferation, and differentiation. Currently, there is no conclusive evidence that human oligodendrocytes have a functional molecular clock. Mouse studies however have shown rhythmic expression of *Bmal1, Per2,* and *Rev-erbα.*^8^ Deletion of *Bmal1* revealed a strong correlation between its expression and OPC proliferation, cell-cycle regulation, and morphology. Moreover, oligodendrocyte-specific genes oscillate throughout the sleep-wake cycle in mice. Therefore, it is hypothesised that human oligodendrocytes contain a functional molecular clock.

Overall, there is mounting evidence indicating that the circadian clock influences crucial processes in glial cell functions. Therefore, disruptions in circadian rhythms can lead to various neurological consequences, which will be further discussed in the following section. Understanding their effects on glial cells can provide insights into maintaining CNS health and developing treatments for related disorders.

## Pathological disruptions of the circadian clock in brain cancer development

The relationship between neurological diseases, such as Alzheimer's or Parkinson's Disease, and dysfunction of the circadian clock is well established.^17^ Additionally, circadian disruption, for instance by perturbations of normal physiological homeostasis due to jet lag or genetic mutations, have been identified as a risk factor for cancer development.^16^ Many cancers show disruption of the circadian clock.^41^ Moreover, there is an association between the loss of circadian control and poor efficacy of anticancer treatments, as well as early mortality among patients with cancer.^16^ Vice versa, an intact circadian clock inhibits proliferation and tumour growth in murine melanoma and colon carcinoma cells.^42^ Still, few studies have investigated the role of the circadian clock in brain cancer development and progression, and these studies primarily focus on glioma. Here, we will highlight key insights from studies on a variety of brain malignancies, broadening the understanding of circadian influences across different types of brain cancer.

### Glioma

The most common group of brain tumours are glioma, comprising over 80% of all brain malignancies.^43^ These heterogeneous tumours are classified in five major groups: adult-type diffuse gliomas, paediatric-type diffuse low-grade gliomas, paediatric-type diffuse high-grade gliomas, circumscribed astrocytic gliomas, and ependymal tumours. Generally, a distinction is made between low-grade and high-grade gliomas based on malignancy. Interestingly, these two tumour classes exhibit contrasting expression levels of the core circadian clock genes. It should be noted that these transcriptomic analyses lack any information about rhythmicity, since the time of sample collection is not included as a factor in the analysis. Therefore, upregulation or downregulation of circadian clock proteins might in fact reflect increased or reduced amplitudes of rhythms. In patients with high-grade glioma, analysis of The Cancer Genome Atlas database reveals that *BMAL1* is upregulated compared to patients with low-grade glioma.^44^ In contrast, combined genomic, transcriptomic, and clinical data analysis unveils a higher mortality in patients with glioma with downregulation of *CLOCK*. Changes in expression of the core clock proteins furthermore influence the internal timekeeping. Studies in a murine model of hypothalamic glioma show that gliomas induce alterations in the circadian clock.^45^ This effect is also observed in a *Drosophila* model, where glioblastoma extends the internal rhythm cycle.^46^

The core clock proteins directly influence glioma progression. For example, glioma stem cell (GSC)-derived CLOCK promotes angiogenesis by interaction with endothelial cells.^47^ GSCs remain a considerable challenge in the treatment of glioblastoma, since they are involved in chemo- and radio-resistance, and immunosuppression.^48^ Interestingly, targeting the circadian clock might improve glioma treatment as GSCs are uniquely sensitive to clock modulation. For instance, GSCs rely on a robust circadian rhythm and downregulation of either *BMAL1* or *CLOCK* through genetic targeting or pharmacological treatment induces cell cycle arrest and apoptosis. Additionally, melatonin can attenuate key signals associated with GSC self-renewal by interfering with the EZH2-NOTCH1 signalling axis.^49^ While BMAL1 and CLOCK are vital for GSC survival, *PER1*, *PER2*, and *PER3* are downregulated in high-grade gliomas.^24^^,^^44^ PER2 suppresses malignant characteristics including proliferation, stemness and invasion of GSCs *in vitro* and *in vivo* by regulating the Wnt/β-catenin and PTEN/AKT/Smad5/Id3 signalling pathways.^50^^,^^51^ In contrast, PER3 promotes astroblastoma progression through the P53/BCL2/BAX signalling pathway and high expression levels of PER3 are correlated with poor overall survival in patients with glioma.^52^ Beyond the altered clock gene expression, intact rhythms in the glioblastoma multiforme cell line T98G during proliferation result in a timed window of vulnerability for the chemotherapeutical agent bortezomib.^53^

Altogether, the exact role of clock genes in glioma remains to be addressed. While some studies show that a functional clock acts as a tumour suppressor,^54^, ^55^, ^56^ there are studies showing that GSCs rely on a robust rhythm.^57^ Also in other cancer types, like in a murine model of acute myeloid leukaemia, tumour cells depend on the circadian clock.^58^ Thus, it may be inferred that the role of the clock as a tumour suppressor is tumour type dependent. All the same, there is a strong body of evidence suggesting that altered expression levels of the core circadian clock genes are crucial for the progression of glioma.

### Rare brain tumour entities

The scarcity of patients or tumour samples of more rare brain malignancies than glioma limits the ability to study the influence of these tumours on the circadian clock. Despite this, there are reports on circadian rhythm disruptions for these brain tumour entities. For example, of patients with untreated primary brain tumours, 59.2% experience insomnia, and 77.7% report poor sleep quality, pointing to circadian disruptions.^59^ For instance, patients with craniopharyngioma, a rare benign parasellar tumour, experience circadian rhythm sleep-wake disorders, which are typically caused by involvement of the SCN and alterations in melatonin transmission.^60^^,^^61^ In medulloblastoma, a brain malignancy occurring primarily in the paediatric population, bioinformatic analysis has revealed that pathways involved in the entrainment of the circadian clock by photoperiod are upregulated.^62^ Choroid plexus tumours exhibit hypermethylated promoter regions of *PER2*, correlating with significantly lower *PER2* expression.^63^ In mice, this downregulation of *PER2* is further associated with increased levels of Cyclin D and Cyclin E, as well as accelerated tumour growth *in vivo*. In patients with untreated pituitary tumours or meningioma, 46.2% and 81.8%, respectively, experience insomnia or poor sleep quality.^64^ Pituitary tumours can also cause a long-term disruption of the sleep-wake rhythms of patients by compressing the optic chiasm, which can also disrupt melatonin rhythms.^65^, ^66^, ^67^ In the same time window, namely in samples collected between 9:00 AM and 12:00 PM, both growth hormone-secreting pituitary adenoma (GHPA) and prolactin-secreting pituitary adenoma (PRLPA) exhibited upregulated PER2 expression, compared to autopsy specimens from deceased individuals.^68^ PER2 promotes pituitary tumourigenesis, and downregulation of PER2 by SR8278, a circadian nuclear receptor REV-ERBα antagonist, protected mice from developing pituitary adenoma.

Taken together, there are strong indications that different brain tumour entities exhibit altered expression of the molecular clock and influence circadian rhythms. There is a growing consensus that the processes driving tumourigenesis, known as ‘cancer hallmarks’, are tightly controlled by the circadian clock in normal cells. However, the role of the circadian clock is generally overlooked in cancer treatment.^7^ Therefore, the next section will delve deeper in the current treatment of brain cancer and explore how the circadian clock influences treatment outcomes.

## Immunotherapy, checkpoints, and other treatments for brain cancer

### Targeting the tumour

Spanning the different types of brain cancer, the main components of treatment in general include maximal safe surgical resection of the tumour, followed by radiotherapy and/or chemotherapy.^18^ Depending on the tumour grade, localisation, and patient age, treatment strategies and therapeutic agents can vary. Treatment outcomes also vary significantly between tumour types. Thus, while certain brain tumours may exhibit favourable prognoses, there remains an urgent demand for novel therapies for high grade brain tumours.

**Fig. 2:**
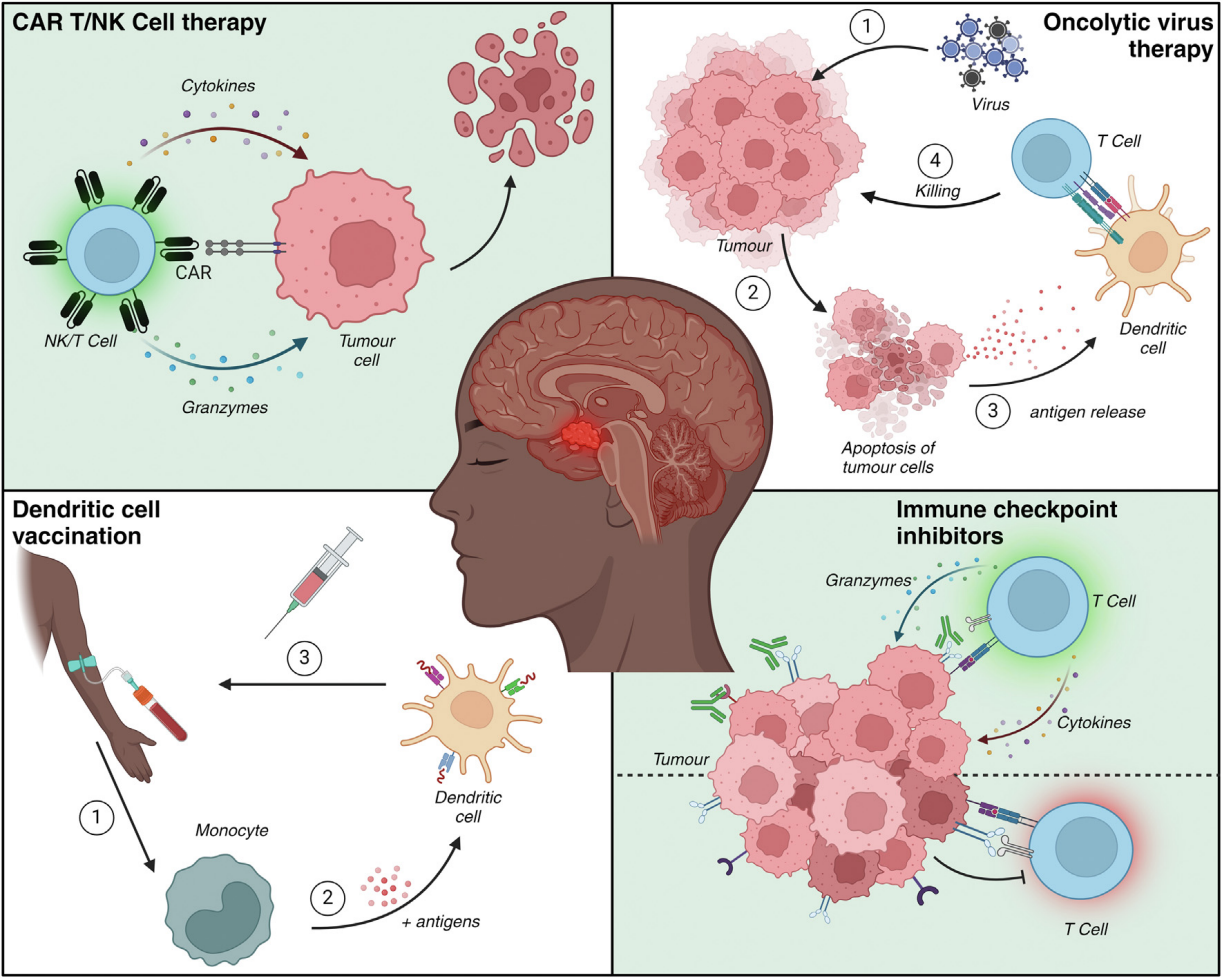
**Immunotherapeutic strategies to target brain cancer.** Several immunotherapeutic strategies have been developed over the past decades, aiming to generate an anti-tumour response from the immune system. Both oncolytic virus therapy and dendritic cell vaccine activate the adaptive part of the immune system via dendritic cells. Oncolytic virus therapy relies on *in vivo* activation of dendritic cells, whereas dendritic cell vaccine therapy involves training dendritic cells *ex vivo* from monocytes. In contrast, CAR T/NK cell therapy uses engineered receptors that enable these cells to precisely target and attack the tumour. Immune checkpoint inhibitor therapy does not focus on training the immune system but instead removes immunosuppression in the TME by blocking one or more inhibitory checkpoints. Made with BioRender. CAR: chimeric antigen receptor; TME, tumour microenvironment.

In recent years, immunotherapy has revolutionised the field of oncology. These therapies aim to activate or boost the immune system to attack and specifically target tumour cells.^20^ Immunotherapeutic strategies include, among others, immune checkpoint inhibitors (ICI), oncolytic viruses, vaccination, and adoptive cellular therapies such as chimeric antigen receptor (CAR)-T cell therapy (Fig. 2).

Immune checkpoints are receptor–ligand interactions between immune cells and their cellular environment, which can be either stimulatory or inhibitory.^69^ These checkpoints balance the immune response to pathogenic infections, thereby preventing excessive activation of immune system. Tumours can exploit the expression of inhibitory immune checkpoint proteins to evade immune surveillance. Immune checkpoint inhibition aims to alleviate the suppression of T cells by blocking the interaction between receptor, located in the membrane of the T cells, and ligand, expressed by the tumour. Monoclonal antibodies are the most commonly used tool for this type of immunotherapy. The blockade of the Programmed Death 1/Programmed Death Ligand 1 (PD-1/PD-L1) immune checkpoint is the most well-known and extensively studied example, demonstrating effectiveness against tumours such as melanoma, renal cell carcinoma and colorectal cancer.^22^ Glioma tumours typically express PD-L1, and higher tumour grades correlate with increased expression levels.^70^, ^71^, ^72^, ^73^ Several studies have trialled the safety and efficacy of blocking the PD-1/PD-L1 interaction in patients with glioma. A single-arm phase II clinical trial (NCT02550249) found that neoadjuvant anti-PD-1 immunotherapy significantly increased median survival time compared to adjuvant anti-PD-1 immunotherapy (417 vs 228.5 days, respectively).^74^ However, in unselected cohorts of patients with glioblastoma, immune checkpoint blockade has not yet improved clinical outcomes.^75^^,^^76^

Although immune checkpoint inhibition has proven to be successful in treating various cancers, the efficacy of antibodies is limited by poor tumour penetration.^69^ Adoptive cell transfer therapies offer an alternative. These therapies often utilise an antigen receptor specific for tumour antigens. To enhance the efficacy of CAR-T cell therapy in brain tumours, intracranial injection can be used to optimise delivery and surpass the requirement to pass the BBB.^77^ Clinical trials for glioblastoma treatment report the preliminary safety and activity of CAR-T cell therapies targeting specific antigens, EGFRvIII, HER2, and IL-13 receptor α2.^78^^,^^79^ Several Phase I and II clinical trials for treating medulloblastoma with either CAR-T or CAR-NK cells are ongoing.^77^ Nevertheless, CAR-T cell therapy is limited by the heterogeneous expression of target antigens by tumour cells, making it difficult to completely eliminate the tumour.^80^

As in CAR therapies, cancer vaccines target tumour antigens to boost anti-cancer immunity. Current therapeutic vaccines include dendritic cell vaccines, where monocytes are stimulated with tumour-associated antigens *ex vivo*, transforming them into dendritic cells.^69^ Upon reinjection into the patient, these cells stimulate and activate cytotoxic T lymphocytes. In the early 2000s, dendritic cell vaccines were already tested in patients with glioblastoma, and many studies showed increased survival. However, recent clinical trials have failed to replicate these results.^77^

Another form of immunotherapy uses oncolytic viruses, designed to selectively replicate in tumour cells, eventually causing their lysis.^69^ This leads to the release of tumour-associated antigens, stimulating anti-cancer immunity. In 2015, the first oncolytic virus therapy became available for melanoma. However, this type of therapy is limited by the intratumoural delivery method, which is particularly costly and difficult for malignant gliomas.^69^

Overall, immunotherapy is a promising type of cancer treatment, especially for brain cancers. Despite its successes, response rates can vary between patients due to, amongst others, the lack of immunogenic antigens or various immune resistance mechanisms.^69^ Immunoregulatory pathways and immune checkpoint expression can be influenced by disruption of circadian rhythmicity in tumour-resident cells to benefit the tumour.^41^ Therefore, future efforts should focus on overcoming the immunosuppression by the TME, as well as identifying new targets that act on important functional pathways of tumours.^81^

### Targeting the clock

In glioblastoma, the circadian clock components are linked to an immunosuppressive TME. BMAL1 and CLOCK activity attracts immunosuppressive microglia, contributing to this environment.^48^ Additionally, the expression of *PER2* suppresses processes like proliferation in glioma and choroid plexus tumours.^24^^,^^44^^,^^63^ Several efforts have been made to target these clock components in glioma. For example, REV-ERB agonists SR9009 and SR9011 have been shown to decrease stemness, survival, migration, and *CLOCK* expression in GSCs, while sparing healthy or differentiated glioblastoma cells.^44^^,^^48^^,^^55^ The synergetic effect of combining clock gene targeting with other therapies, as described in glioblastoma *in vitro*, likewise holds promise.^82^

Alternatively, reducing *BMAL1* expression using CRY stabilisers such as KL001 or SHP656 has also shown potential in targeting GSCs.^57^ Interestingly, the efficacy of SR9009 and SR9011 in mouse models was comparable to that of temozolomide treatment in patient-derived xenograft glioblastoma models, while evincing reduced toxicity.^7^ These findings indicate that targeting the clock in glioma presents a promising prospective for treatment. To our knowledge, these compounds have not been tested on other brain cancer entities, however, given their impact on glioma cells, they might alter the TME in a way that enhances the efficacy of immunotherapeutic therapies.^48^ Illustrating the potential of targeting the clock is a pioneering study in a glioma mouse model, which demonstrates that combining REV-ERB agonists with anti-PD-1 treatment yields a synergistic effect, significantly extending survival.^48^ Forthcoming research ought to focus further on the interplay between circadian clock components and immunotherapy, revealing potential synergies in treatment.

## Circadian orchestration of immune cell dynamics

Following circadian rhythms, the human immune system exhibits heightened sensitivity during daytime when environmental challenges are most apparent, while reducing the chance of detrimental immunity when challenge is diminished at night.^83^ This heightened sensitivity in the day is firstly illustrated by the migration of leukocytes to peripheral organs at the onset of the behavioural active phase.^84^ The time-of-day-dependent expression of migration factors on immune cells drives their specific localisation to tissues. For example, dendritic cells exhibit rhythmic migration into lymphatics, enforcing specific timepoints for efficient T cell activation.^85^^,^^86^ Furthermore, lymphocytes demonstrate time-dependent differences in infiltration into the tumour site, with peak infiltration occurring during the behavioural active phase in mice.^87^ Anti-tumour immunity is thus shaped by the prompt trafficking of immune cells during the active phase. In conjunction with diurnal oscillations in the permeability of the BBB, immunity within the brain is under strict circadian regulation.^88^^,^^89^

In keeping with localisation, immune cells show timed variations in function. This includes antigen processing, cytokine release and cytolytic factor expression, as well as diurnal oscillations in polarisation of T cell, and macrophage signatures.^83^^,^^86^^,^^87^^,^^90^ Co-stimulatory factor expression is also under circadian control. In dendritic cells oscillations in CD80 expression directly affects CD8+ T cell activation and therefore cancer immunosurveillance.^91^ Consequently, time may affect the efficacy of anti-cancer vaccines and feasibly other immunotherapies. A key factor herein is the rhythmic regulation of the immune component of the TME. In the brain, circadian oscillations in microglial inflammatory cytokine expression could affect local cancer immunity in a clock-controlled manner.^35^ The clock furthermore enforces diurnal changes in immune checkpoint expression on CD8+ T cells and myeloid-derived suppressor cells, thus timely affecting the efficacy of immune checkpoint inhibition and CAR T cell therapy in murine cancer model systems.^87^^,^^92^

Clinically, the oscillating expression of PD-1 on tumour-associated macrophages underlies the time-of-day dependent variation in ICI efficacy in patients with melanoma.^93^^,^^94^ ICI proves most effective when administered in the morning, thereby interfering during the time at which the macrophages most strongly inhibit T cell activation. The potential benefit of circadian targeting in cancer is studied in multiple clinical trials, of which some notable trials are portrayed in Table 1.

**Table 1:** Clinical trials investigating the influence of circadian rhythms on cancer treatment.

Trial ID/Registration	Title	Phase	Cancer type (stage)	Intervention(s)	Start date	Completion date	Study type
**Breast cancer**							
NCT04864405	Evaluating the Dose Timing (Morning vs Evening) of Endocrine Therapy and Its Effects on Tolerability and Compliance	4	Breast Cancer	Morning vs evening endocrine therapy	30-06-2021	29-07-2023	Interventional
NCT04401189	The Role of Circadian Rhythms in Cancer-Related Symptoms (CHRONO)	N.A.	Breast Cancer	Circadian rhythms and CRS before vs after treatment (surgery/chemotherapy)	01-06-2020	06-2022^a^	Observational
NCT03205033	Melatonin as a Circadian Clock Regulator, Neuromodulator and Myelo-protector in Adjuvant Breast Cancer Chemotherapy	2	Breast cancer	Melatonin 7 days before until 21 days after chemotherapy	01–2016	01–2017	Interventional
NCT03217201	Systematic Light Exposure for Fatigue in Breast Cancer Patients	N.A.	Breast cancer	Effect of SLE on CRF	25-01-2018	26-1-2022	Interventional
NCT02954809	Effects of Bright Light Therapy on Fatigue, Sleep and Circadian Activity Rhythms in Lung Cancer Survivors	N.A.	Breast cancer survivors	Morning bright light therapy vs dim light.	10-2016	05-2017^a^	Interventional
NCT06418139	Association of Pembrolizumab Infusion Time and Efficacy in Patients With Non-metastatic Triple-negative Breast Cancer (TNBC) Treated With Neoadjuvant Chemotherapy and Immunotherapy (PEMCLOCK)	N.A.	Non-Metastatic Breast Carcinoma (TNBC)	N.A.	05-2024^a^	09-2026^a^	Observational
**Colorectal cancer**							
NCT03955510	Abnormal Food Timing and Circadian Dyssynchrony in Alcohol Induced Colon Carcinogenesis (AFT)	N.A.	Colorectal cancer	Effect of timing of food intake and alcohol use on susceptibility to colorectal cancer.	31-07-2016	30-11-2024^a^	
NCT00852228	Optimal Control of Liver Metastases From Colorectal Cancer With Cetuximab and Hepatic Artery Infusion of Chemotherapy (OPTILIV)	2	Liver metastases from colorectal cancer	Chronomodulated vs conventional chemotherapy	07-2008	2015-12^a^	Interventional
**Head and neck cancer**							
NCT05083416	Effect of Prolonged Nightly Fasting on Immunotherapy Outcomes in HNSCC - Role of Gut Microbiome		Head and neck cancer	PNF vs no PNF effect on immunotherapy	20-10-2021	24-05-2024^a^	Interventional
NCT03196869	the Study of Effect of Chronomodulated Chemotherapy on the Dendritic Cells Subsets in the Treatment of Advanced Nasopharyngeal Cancer	2	Locally Advanced HNSCC	Chrono-chemotherapy vs routine chemotherapy	7-4-2014	12-8-2022^a^	Interventional
**Lung cancer**							
NCT05637580	Pathological Tumor and Lymph Node Responses After Neoadjuvant Immunochemotherapy in Initially-unresectable NSCLC	N.A.	NSCLC	N.A.			Observational
NCT04650490	SRS Timing With Immune Checkpoint Inhibition in Patients With Untreated Brain Metastases From Non-small Cell Lung Cancer (STICk-IM-NSCLC)	2	NSCLC with brain metastasis	Timing of stereotactic radiosurgery relative to immunotherapy	03-2023^a^	03-2026^a^	Interventional
NCT05988970	Impact of Circadian Rhythm on the Spread of Circulating Tumor Cells in Lung Cancer Patients	N.A.	Lung cancer	CTC before and after treatment	29-1-2024	09-2025^a^	Interventional
**Brain cancer**							
NCT04669574	Assessing Sleep and Circadian Rhythms in Primary Brain Tumors Patients	N.A.	Primary brain tumour	Detect sleep disturbances in patients.	29-06-2021	01-01-2025^a^	Observational
NCT02781792	Temozolomide Chronotherapy for High Grade Glioma	2	High grade glioma (II-IV)	TMZ morning vs evening administration	11-08-2016	14-07-2024	Interventional
**Other**							
NCT04827446	Lighting Intervention for Cancer-related Fatigue	N.A.	Breast and prostate cancer, HSCT receivers.	Effect of light levels on cancer related fatigue.	15-7-2021	09-03-2023	Interventional
NCT02937519	Chronomodulated Chemotherapy Followed by Concurrent Chemo-radiotherapy With IMRT in the Treatment of Advanced Nasopharyngeal Cancer	2	Oesophageal Neoplasms	Chrono-chemotherapy vs routine chemotherapy	06-2015	06-2018^a^	Interventional
NCT05737732	The Ambient Light Multiple Myeloma Study	N.A.	Multiple myeloma	Effect circadian effective vs ineffective lighting on patients receiving ASCT	13-02-2023	30-06-2027^a^	Interventional
NCT02187315	Induction Chemotherapy Followed By Chrono-chemotherapy Concurrent With IMRT Of Locally Advanced NPC Clinical Study	2	Nasopharyngeal carcinoma	Chrono-chemotherapy vs routine chemotherapy	05-2014	12-2019^a^	Interventional
NCT05511740	Circadian as A Prognostic Factor For Radiation Response in Cervical Cancer	N.A.	Cervical cancer	Morning vs evening radiation therapy	01-2010	07-2014	Interventional

ASCT: autologous stem cell transplant; CRF: cancer related fatigue; CRS: cancer-treatment related symptoms; CTC: circulating tumour cells; HNSCC: head and neck squamous cell carcinoma; HSCT: hematopoietic stem cell transplant; NSCLC: non-small cell lung carcinoma; PBT: primary brain tumour; PNF: prolonged nightly fasting; SLE: systematic light exposure; TMZ: temozolomide; TNBC: triple-negative breast cancer.

a Expected completion date.

Beyond identifying pathways worth exploring for time-specific targeting, the role of the molecular clock pathway itself has gained interest in the context of tumour immunity. While the immune system throughout the body adheres to a circadian rhythm, within tumours the clock genes can exert different functions. Clock gene-enriched pathways are directly linked to immune evasion in various cancers.^90^ Disruption of circadian rhythmicity in tumour-resident cells can influence immunoregulatory pathways and immune checkpoint expression to the cancer's advantage, marking the significance of the molecular clock in cancer immunity.^41^ In glioblastoma multiforme, *CLOCK* expression is correlated with higher microglial presence and reduced activation of CD8+ T cells in a non-diurnal manner.^48^ Likewise, the upregulation of chemoattractant olfactomedin-like 3 (OLFML3) by *BMAL1* and *CLOCK* facilitates the migration of immunosuppressive microglia into the glioblastoma TME.^95^ Blockade of the CLOCK-OLFML3 pathway is currently being studied as a potential target for GBM.^96^ Understanding of the circadian orchestration of tumour-immune cell dynamics in brain cancer paves the way for improved chronotherapeutic strategies in immunotherapy.

## Future perspectives for brain cancer treatment

### The timing of treatments or surgical interventions affects outcomes and side effects

It has become apparent that timing of treatments or interventions can affect the outcomes of various pathologies. For instance, timing antihypertensive drugs to circadian rhythms can prevent the morning peak in cardiovascular disease incidence, and scheduling aortic valve replacement surgeries in the afternoon is associated with a reduced incidence of major adverse cardiac events.^97^ Moreover, increasing evidence shows that chrono-modulation of chemotherapy reduces toxicity without compromising efficacy.^98^ In the case of glioma, a recently published systematic review describes that temozolomide cytotoxicity is highest during the peak of BMAL1 expression, which occurs in the morning.^99^ Another study showed that daily rhythms of O^6^-Methylguanine-DNA Methyltransferase (MGMT) expression determine the phase-dependent sensitivity of glioblastoma cells to temozolomide.^100^ The methylation status of *MGMT* has a strong prognostic value for treatment with temozolomide. Therefore, the rhythmicity of the *MGMT* methylation should be considered when analysing biopsies, to further enhance the prognostic value of temozolomide treatment.^100^ Next to drug toxicity and prognostic value, therapy efficacy is apt to be controlled temporally, and tumour growth rates can be impacted differently based on the timing of treatment administration.^54^ Although more clinical data is needed to solidify these findings, adjusting the timing of the treatment is seemingly a feasible and easily implemented strategy. It can be argued that aligning immunotherapy to circadian rhythms might similarly benefit the outcomes of brain cancer treatment. Therefore, the next section further elaborates on this opportunity.

### Chrono-immunotherapy and circadian principles for development of new and improved treatments for brain cancer

**Fig. 3:**
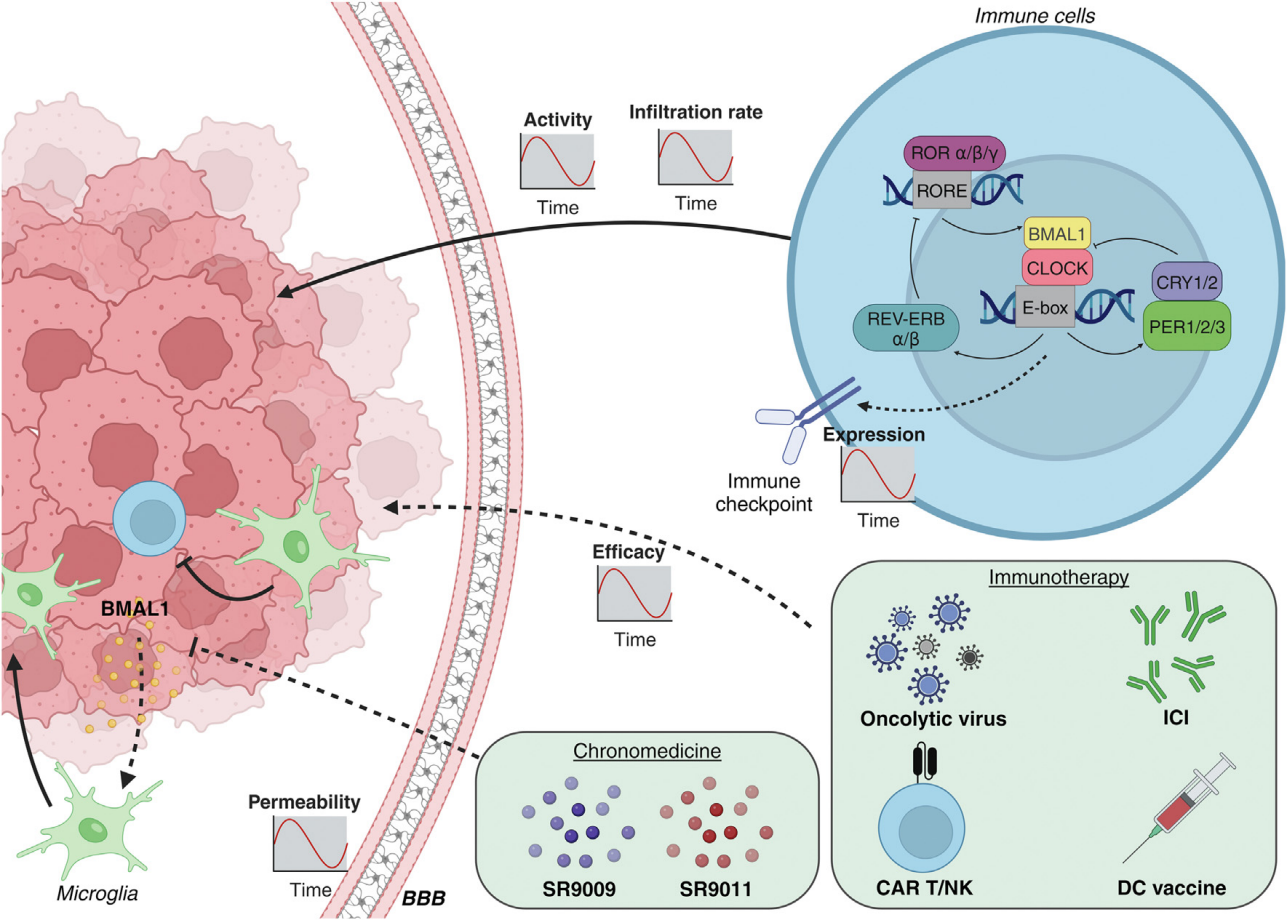
**Integration of circadian rhythms and immunotherapy for enhanced treatment of brain tumours.** Circadian rhythms dictate various processes that affect the efficacy of brain cancer treatment. The immune cells exhibit circadian changes in activity, sensitivity, immune checkpoint expression, and infiltration rates. Similarly, circadian rhythms dictate the permeability of the BBB. On the other hand, the malfunctioning of the circadian clock in brain cancer boosts cancer progression. Simultaneously, these changes can attract immunosuppressive microglia, further shaping an immunosuppressive TME. Taken together, circadian rhythms may provide an optimal time-window for administering chronomedicine, targeting the clock, or immunotherapeutic interventions. Made with BioRender. BBB: blood-brain barrier; CAR: chimeric antigen receptor; DC: dendritic cell; ICI: immune checkpoint inhibitors; NK: natural killer; RORE: ROR response element; TME: tumour microenvironment.

Circadian cues modulate immunity significantly dependent on the time of day. The broad impact of circadian rhythms in brain cancer is embodied by the oscillating infiltration and function of immune cells in the TME, the synchronised permeability of the BBB and thus accessibility of brain tumours for therapy, as well as a non-diurnal role for molecular clock genes on cancer immunity (Fig. 3). How these factors coalesce will unveil a more fundamental understanding of brain malignancies and will uncover the potential benefit of chronotherapy as well as targeting of the clock-immunomolecular circuitry (Table 2).

**Table 2:** The brain tumour's time table.

Circadian modulation of …	Cross-cancer discoveries	Pending brain cancer queries
**Brain cell activity**	▪ Circadian rhythms influence neuronal activity and glial cell functions.^8^ ▪ Disruption of circadian rhythmicity described as a carcinogen.^16^	▪ The role of circadian genes and their disruption as propagators of malignant transformation of neural cell types. ▪ Temporal dynamics of the interaction between brain cells and immunity.
**Tumour cell phenotype**	▪ Circadian disruptions and clock alterations foster cancer initiation and regulate cancer hallmarks.^23^ ▪ Tumour cell migration and metastatic potential are regulated by circadian rhythms.^23^ Disruption of circadian rhythms can enhance metastatic behaviour.^101^ ▪ Circadian genes impact brain tumour proliferation, metabolism, and angiogenesis independent of diurnal oscillations.^16^^,^^47^^,^^57^	▪ Specific mechanisms effectuating circadian oscillations in tumour phenotype to reveal potential targets. ▪ The integrity of the tumour clock and the impact of altered clock gene expression on characteristics of different brain cancer types. ▪ Comprehensive profiling of circadian changes in immunotherapy target antigen and immune checkpoint expression.
Blood-brain barrier (BBB)	▪ Circadian rhythms regulate BBB permeability, temporally affecting e.g., drug delivery.^88^^,^^89^ ▪ BBB integrity is challenged by circadian disruption.^88^ ▪ BBB integrity is modulated by brain cancer.^102^^,^^103^	▪ The timed accessibility of brain tumours to immunity and (cellular) immunotherapy. ▪ Brain cancer dissemination and the formation of brain metastases following circadian oscillations in BBB permeability.
Tumour-immune microenvironment (TIME)	• Tumour immune cell infiltration exhibits circadian variation.^87^ ▪ Disruption of the clock associates with immune cell exhaustion.^41^ ▪ Molecular clock genes can reshape the brain cancer TME to be more immunosuppressive and pro-proliferative.^95^^,^^104^	▪ Circadian control of the infiltration into and immune status of cells in the brain TME. ▪ Circadian–immune pathway interplay, identification of clock-driven mechanisms in immunosuppression and tumour progression. ▪ Potential of synergetic targeting of the clock and cancer immunity.

In terms of chronotherapy, synchronising treatment with circadian rhythms has the potential to enhance immunotherapy efficacy. To this and the patients' advantage, patients’ sleep-wake cycles and the entrainment of the clocks (e.g., light/dark cycles, food intake timing, influence of electronic devices) should be guarded. The benefit of administration of immunotherapy at specific times throughout the day can be studied quite inexpensively in the clinic. This would arguably be most beneficial in tumours that exhibit an intact circadian rhythm, such as in glioblastoma, as these tumours may display timed vulnerability to cancer immunity.^57^ Investigating the integrity of the circadian clock in different forms of brain cancers might elucidate timed variation in susceptibility. This is relevant for all cell types residing in the TME, as their timed variation in immunosuppressive qualities jointly contributes to the timeframe of vulnerability of the tumour to immunity. Within the current understanding, targeting would be most favourable at a time when the BBB is most permeable, immune effector cells readily infiltrate the tumour site, and tumour cells themselves present minimal resistance. The circadian immune status of, e.g., microglia and tumour associated macrophages furthermore affects both effector cells and tumour cells in a timely manner. Delving into the circadian rhythms of the brain TME promises to unveil the optimal timing for administering immunotherapy.

Still, circadian rhythmicity is disturbed in many cancers.^41^ This sets the stage for more precise targeting of the molecular clock. Since disruption of the clock is connected to immune evasion, its restoration possibly relieves this effect to improve immunotherapy outcomes. However, selective restoration may prove complicated by the ubiquitous expression of the clock genes. It is therefore imperative to unravel targets in the clock gene - immunoregulatory pathway interaction. This calls for a more fundamental study of the pathways by which molecular clock genes affect immunosuppression in brain cancer and the TME. Furthermore, combining immunotherapy with the targeting of the clock could ultimately increase the potency of immunotherapy in brain cancer. Thus, integrating circadian rhythms, the molecular clock, and immunotherapy can greatly benefit brain cancer treatment.

## Conclusion

Circadian rhythms intricately govern numerous cellular processes. While in healthy cells circadian rhythms orchestrate activity in a controlled manner, in cancer, alterations in molecular clock expression often fuel tumour progression by fostering the survival and proliferation of malignant cells. These changes further bolster the tumour's ability to establish an immunosuppressive TME. Although various immunotherapeutic therapies have been tested for brain cancer, their success remains limited. A pivotal determinant of immunotherapy outcomes is the circadian rhythm governing the immune system and TME. Strategically targeting and harnessing the circadian clock for brain cancer treatment has the potential to revolutionise therapeutic approaches and significantly enhance the efficacy of immunotherapy.

## Outstanding questions

Knowledge on the role of the circadian clock in brain cancers remains largely limited to glioma. Enhancing our understanding of the circadian clock in all forms of brain cancer is essential for improving treatment. While chronotherapy for glioma is still in its infancy, current studies are paving the way for future advancements in brain cancer therapy. With the increased availability of Whole Genome or Whole Exome Sequencing data (WGS/WES), larger cohorts of patient data are emerging, providing a valuable resource for research. Since the circadian clock causes changes in expression throughout the 24-h cycle, the collection of data might mask rhythmic changes. Therefore, we recommend that sequencing data should include timestamps for the time of collection to enable bioinformatic analyses based on time. This will provide crucial information for the further development of chronotherapy and immunotherapy for brain cancer. Furthermore, the profound influence of circadian rhythms in the immune system as well as the TME urges further research into these mechanisms in brain cancer. Incorporating the dimension of time into clinical decision-making for immunotherapy can ultimately enhance therapy outcome, making circadian rhythms a critical factor in the design and investigation of brain cancer treatments.

## Contributors

MQ, MvO and SC contributed to the concept and design of this review. MQ and MvO performed the literature search and reviewed and selected the papers for inclusion. MQ, MvO and SC wrote and edited the manuscript. MQ and MvO created the figures. SC, LWvL and NB critically revised the manuscript, the figures and tables. All authors read and approved the final version of the manuscript.

## Declaration of interests

MQ, MvO, SC, NB: None. LWvL: Outside the current work: consultancy fees to UMCU from Abbott, Medtronic, Vifor, Novartis.
